# Diabetic patients’ perspectives on the challenges of glycaemic control

**DOI:** 10.4102/phcfm.v7i1.767

**Published:** 2015-07-30

**Authors:** Oladele V. Adeniyi, Parimalane Yogeswaran, Graham Wright, Benjamin Longo-Mbenza

**Affiliations:** 1 Department of Family Medicine, Faculty of Health Sciences, Walter Sisulu University, Mthatha, South Africa; 2 Centre for Health Informatics Research and Development (CHIRAD), University of Fort Hare, South Africa

## Abstract

**Introduction:**

The factors affecting the control of diabetes are complex and varied. However, little is documented in the literature on the overall knowledge of diabetic patients about glycaemic control. This study explored the patients’ perspectives on the challenges of glycaemic control.

**Methods:**

In this qualitative study, semi-structured interviews were conducted with seventeen purposively selected diabetic patients with HBA1c ≥ 9% at Mthatha General Hospital, South Africa. The interviews were conducted in the isiXhosa language and were audiotaped. Two experienced qualitative researchers independently transcribed and translated the interviews. Thematic content analysis was conducted.

**Results:**

Three main themes emerged: overall knowledge of diabetes and treatment targets, factors affecting the control of diabetes and how glycaemic control could be improved.

The majority of the participants demonstrated poor knowledge of treatment targets for diabetes. The majority of the participants reported that lack of money affected their control of diabetes. Some of the participants reported that the nearest clinics do not have doctors; hence, they are compelled to travel long distances to see doctors.

**Conclusion:**

Poverty, lack of knowledge and access to doctors affect the control of diabetes in the rural communities of Mthatha, South Africa. The government should address recruitment and retention of doctors in primary health care.

## Abstract (fr)

**Perspectives des patients diabétiques sur les défis du contrôle de la glycémie.**

**Introduction:**

Les facteurs affectant le contrôle des diabétiques sont complexes et variés. Cependant, il y a peu de choses dans la littérature sur les connaissances générales des patients diabétiques sur le contrôle de la glycémie. Cette étude a examiné les perspectives des patients sur les défis du contrôle de la glycémie.

**Méthodes:**

Pour cette étude qualitative, on a mené des entrevues semi-structurées avec 17 diabétiques choisis expressément avec le HBA1c ≥ 9% à l’hôpital général de Mthatha, en Afrique du Sud. Les entrevues ont été effectuées en langue Xhosa et enregistrées. Deux chercheurs qualitatifs expérimentés ont transcrit et traduit séparément les entrevues. Puis une analyse du contenu thématique a été effectuée.

**Résultats:**

Trois thèmes principaux sont apparus: la connaissance générale sur le diabète et les objectifs à atteindre en matière de traitement, les facteurs affectant le contrôle du diabète et comment améliorer le contrôle de la glycémie.

La majorité des participants avait peu de connaissances des objectifs à atteindre pour le traitement du diabète. La majorité des participants a déclaré que le manque d’argent affectait leur contrôle du diabète. Certains participants ont déclaré que les cliniques les plus proches n’avaient pas de médecins; aussi, étaient-ils obligés de se déplacer loin pour consulter des médecins.

**Conclusion:**

La pauvreté, le manque de connaissances et l’accès aux médecins affectent le contrôle du diabète dans les communautés rurales de Mthatha, en Afrique du Sud. Le gouvernement devrait s’occuper du recrutement et du maintien en poste des médecins dans le domaine de la santé primaire.

## Introduction

Diabetes mellitus is a chronic non-communicable disease that affects about 2 million people in South Africa.^1^ According to the International Diabetes Federation, an estimated 63 061 South Africans died from diabetic-related complications in 2012.^2^ The increase in the prevalence of diabetes mellitus (predominantly type 2) in the population is the cause of the rise in complications: non-traumatic amputation, cardiovascular diseases, blindness, end-stage renal failure, and many others.^3,4,5,6,7^ Poor glycaemic control amongst patients with diabetes mellitus constitutes a major public health problem.^8^ The progression of diabetes complications occurs due to poor glycaemic control, which can be managed by quality healthcare services.^9^

Diabetic patients should be empowered with knowledge to manage themselves.^10,11^ Basic knowledge of diabetes is considered a prerequisite for self-care management.^11^ Diabetes self-care management has been linked with diabetes education and knowledge acquisition.^10, 12^ Self-care management is associated with a reduction of complications and improvement in the quality of life of diabetic patients.^13^

The depth of diabetic information that would lead to better glycaemic control is not documented in the literature. However, patients should know about the nature of diabetes, complications, medication and side-effects, role of dietary adjustment, exercises, self-monitoring of blood sugar, treatment targets and many others. This should start at the time of diagnosis and be updated at regular intervals. The benefits of maintaining normal body mass index must be explained. Patients need to understand the deleterious association of cigarette smoking and cardiovascular risks. Patients should be taught how to take care of their feet, inject insulin, recognise complications and skills required to cope with living with diabetes.^5^

Diabetes education should be evidence-based and structured according to the socio-demographic characteristics of each patient.^10^ According to Kumar and Clark, if health care workers fail to provide appropriate information, then friends and family members give patients all sorts of inaccurate information.^5^ Diabetic educators are crucial to the successful implementation of diabetic education programmes.^8^ However, very few public health care facilities in South Africa can boast of diabetic educators;^10^ therefore, time to educate patients is limited given the vast numbers of patients and shortage of health personnel.

The importance of the knowledge of diabetes to glycaemic control has been evaluated in a number of studies.^14,15,16,17,18^ There have been mixed reports in the literature: whilst some studies reported that an improvement of knowledge of diabetes might predict good glycaemic control,^8,19^ others demonstrated no significant association with glycaemic control.^11,16,20^ Earlier studies suggested that patients with chronic diseases (T2DM inclusive) who are active participants in their health care have better health outcomes.^14,15^ Heisler et al. highlighted the American Diabetes Association's position to launch a campaign to urge diabetic patients to be aware of their treatment target and actual values of HBA1c, blood pressure and cholesterol levels (their ABCs).^16^

A number of studies have examined the relationship between knowledge of treatment target and glycaemic control.^16,17,18^ The majority of the participants in these studies had no knowledge of their recent HBA1c.

Heisler et al. concluded that knowledge of HBA1c alone was not sufficient to translate increased understanding of diabetes care into improvement in self-management of diabetes.^16^ Santos et al. reported that glycaemic control did not correlate with knowledge of diabetes amongst the participants in their study.^11^ They suggested that theoretical or practical understanding of diabetes is not by itself significantly associated with appropriate glycaemic control.

Iqbal et al. examined the impact of improving the knowledge of diabetic patients on glycaemic control.^19^ The baseline measurement of the knowledge of the participants showed that the majority were not familiar with HBA1c. Knowledge of glycaemic control was generally poor amongst the participants. Intervention with diabetic education yielded improvement in glycaemic control amongst poorly controlled T2DM, who were in the unfamiliar group (10.7% versus 9.5%, *p* = 0.04). Knowledgeable diabetic patients tend to have a good attitude, which is linked to improvement in glycaemic control.^8^

A few studies found no significant association between the level of knowledge of diabetes and glycaemic control.^11,20^ Notwithstanding, there is overwhelming evidence that diabetes education is central to self-care management and ultimately, improvement in glycaemic control.^5,10,19,21^ Hence, the Joint Task Force of American Diabetes Association, the European Association on the Study of Diabetes, (2012)^21^ and the Society of Endocrinology Metabolism and Diabetes of South Africa Guideline, (2012)^10^ recommended diabetic education as a major component of the care for diabetic patients.

Many studies examined the association and magnitude of the relationships between health literacy and diabetes outcomes. However, qualitative exploration of the depth of knowledge of patients about glycaemic control appears to be neglected in the literature. Such information might influence the structure of patient education by clinicians. The feedback from patients provides valuable inputs into quality improvement of health care services, policy formulation and guideline development.

## Method

### Operational definitions

Good glycaemic control: According to the Society of Endocrinology, Metabolism and Diabetes of South Africa, the majority of patients with a glycosylated haemoglobin level < 7% will be considered as having achieved good glycaemic control. This is also supported by the recommendation of the American Diabetes Association.^21^ HBA1c levels above 7% will be regarded as poor glycaemic control in the study.

Critically poor glycaemic control: Levels of glycosylated haemoglobin ≥ 9% is considered to be critically poor in this study.

Rural versus semi-urban community: there is no consistency in the definitions. However, South African government policies refer to rural areas as those that are non-metropolitan.^22^ They are characterised by inferior infrastructure, low income, poor site conditions, unreliable water availability and poor access to health facilities.^23^ Rural areas in South Africa have been defined in relation to poverty, underdevelopment and low habitation.^24^ The place of residence of participants, other than Mthatha, is classified as rural in this study.

Semi-urban community: based on the pace of urban population growth of rural communities, semi-urban areas share the characteristics of rural-urban communities. Mthatha is considered to be semi-urban in this study.

### Aim

The aim of this study was to explore the overall knowledge of diabetic patients about diabetes and glycaemic control. It was the second component of a bigger study on diabetes in rural South Africa.

### Setting of the study

The study was conducted at Mthatha General Hospital, Mthatha, Eastern Cape Province, South Africa. This is a 258-bed district hospital serving a predominantly Xhosa-speaking population of about 1.5 million people.

### Study design

In order to gain an in-depth understanding of the patients’ perspectives of diabetes and glycaemic control; a qualitative study was conducted using semi-structured open-ended interviews with prompts.

### Period of study

The study was conducted in October and November 2013.

## Research methods and design

### Sampling and procedure of the study

Seventeen purposively selected participants were drawn from the follow-up review of results of participants who took part in the first component, which was a quantitative study. Critical case sampling was employed to track down patients with the worst control of diabetes and high risks for diabetes complications. Participants were selected if their recent glycosylated haemoglobin was ≥ 9%, they were willing to participate in the interview, age ≥ 30 years at diagnosis of DM and had been on treatment for diabetes for a minimum period of at least one year. Participants were excluded from the interview if they were receiving treatment for less than one year or acutely ill at the time of the study.

The interview explored the following key areas of diabetes and glycaemic control: nature of diabetes, complications, treatment targets, medication and access, adherence to treatment and self-care efforts to achieving control.

A trained interviewer used open-ended techniques to elicit in-depth information from the participants. An interview guide was used to ensure that the key questions were asked if they did not arise spontaneously. The interviews were conducted in the local language of the participants (isiXhosa) to ensure that participants were free and confident in their responses. The interviews were audiotaped and the interviewer also kept notes of the process. Recruitment continued until no new information emerged during the interviews (data saturation).

**Data analysis**: Two experienced qualitative researchers transcribed the audiotaped interviews independently and translated them verbatim. Notes were then compared to ensure accuracy of transcription and translation. Field notes were reviewed for additional information. Thematic analysis technique was used for data analysis. Line numbers were used to identify questions asked by the interviewer and responses made by the participants. Themes were developed from the participants’ responses on different questions and various issues. Participants’ responses were categorised according to themes. Themes were colour-coded and those colours were used to shade any response relating to specific themes in the interviews. Content theme analysis was employed to maximise the chance that all relevant information was grouped and coded appropriately. The notes were cross-checked to ensure responses of participants were coded appropriately.

## Ethical considerations

Institutional approval: the researchers obtained ethical approval from the Walter Sisulu Higher Degrees and Biosafety and Ethical Committee (Protocol number: 031/2013; Dated on 09/10/2013), Nelson Mandela Hospital Complex Ethics committee and the Eastern Cape Department of Health Epidemiological Research and Surveillance Management. Consent of the Chief Executive Officer of Mthatha General Hospital was sought and obtained. Each participant provided written informed consent after obtaining relevant information on the process of the research. They were each given a participant's information sheet detailing the purpose, process, who to contact and dissemination of research output.

## Results

Characteristics of Participants (Table 1): according to the gender, age, residence, employment status, duration of diabetes and glycosylated haemoglobin. The majority of participants were women (11/17), unemployed (10/17) and lived in rural communities (14/17). Four participants were pensioners (4/17) and 3 participants were employed. The ages of the participants ranged from 45–72 years. The duration of type 2 diabetes mellitus amongst the participants were: 2–5 years (8/17), 6–10 years (3/17) and > 10 years (6/17). The levels of control of diabetes amongst the participants were critically poor (HBA1c ≥ 9%) with a range of 9.4%–13.6% and mean HBA1c of 11.4%.

**TABLE 1:** Characteristics of participants.

Participants	Gender	Age (years)	Residence	Employment status	T2DM duration (years)	HbA1c (%)
P01	F	56	Rural	Unemployed	7	10.8
P02	F	65	Rural	Unemployed	15	9.4
P03	F	70	Rural	Pensioner	> 20	12.4
P04	M	53	Semi-urban	Employed	7	9.6
P05	F	48	Rural	Unemployed	3	12.8
P06	F	54	Rural	Unemployed	2	11.0
P07	M	66	Rural	Unemployed	> 10	13.6
P08	F	45	Rural	Employed	3	10.4
P09	M	51	Semi-urban	Unemployed	5	10.8
P10	F	68	Rural	Pensioner	> 15	9.4
P11	F	63	Rural	Unemployed	8	12.6
P12	M	50	Rural	Unemployed	5	13.2
P13	F	42	Rural	Unemployed	2	11.2
P14	M	55	Rural	Employed	4	9.8
P15	M	62	Rural	Pensioner	> 10	10.5
P16	F	72	Semi-urban	Pensioner	> 20	13.6
P17	F	54	Rural	Unemployed	3	12.4

P, participants; M, males; F, females; T2DM, Type 2 Diabetes Mellitus; HbA1c, Glycosylated haemoglobin.

### Themes

**FIGURE 1:**
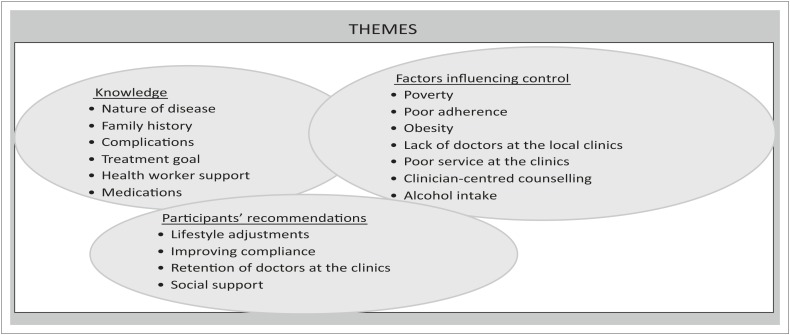
Pictorial analysis of the themes and sub-themes.

Three main themes emerged from the interviews: knowledge of diabetes and glycaemic control amongst participants, factors influencing the control of diabetes and perspective of participants on how to improve glycaemic control. The themes and sub-themes are presented in Figure 1.

### Theme 1: Knowledge of diabetes and glycaemic control amongst participants

All the participants understood the nature of the disease: incurable but manageable. The majority of the participants (*n* = 15/17) had at least one family member already diagnosed with diabetes. They confirmed that diabetes could affect family members:

‘My mother was living with diabetes and it affected her eyes. I was diagnosed of diabetes about three years ago.’ (Participant 05; F, 48 years)

There was a good level of awareness of diabetes complications amongst the participants:

‘I developed stinking wounds, filthy lump under my foot and spread to the leg, the doctor told me that I have few days to live before I die, if the leg is not amputated.’ (Participant 07; M, 66 years)

‘Not dating any woman because of this diabetes, my body is not responding anymore, I used to be strong but has lost my spark, I went to the pharmacy to buy boosters but they are not helping.’ (Participant 12; M, 50 years)

‘I have something that developed few weeks ago, my feet are feeling hot and painful, and when asleep I take them out of the blankets.’ (Participant 16; F, 72 years)

The majority of the participants (*n* = 13/17) had no knowledge of what is considered to be good glycaemic control or the desired treatment target for using diabetic medication. The minority (*n* = 4/17) who had some idea about the control of blood sugar reported that they were not sure if blood sugar levels should be less than 8 or 10:

‘My blood sugar fluctuates between 20, 17 and 18, only last month; I saw a change when it was 6.8. I am not sure what the level is supposed to be.’ (Participant 08; F, 45 years)

‘My sugar is not controlled because I faint regularly and wake up in the hospital then feel better. The doctor placed me on insulin injection. I think the sugar should be less than 10 if it is normal.’ (Participant 17; F, 54 years)

The majority of the participants (*n* = 14/17) get help and support from family members. The doctors and nurses also provide assistance to the diabetic patients:

‘I always get a good service at the hospital because I make sure that I get to the clinic first and I always meet the health care workers in good mood.’ (Participant 10; F, 68 years)

Only a few participants (*n* = 7/17) reported that they do not get the help they need from the doctors and nurses. They expressed their disappointment in the quality of care the doctors and nurses offered at the hospital; reference was made to the short consultation time, lack of listening during consultation, eagerness by doctors to write medication and the long queues:

‘The healthcare workers care less about the patients but I understand them because they care for 200 patients in a day.’ (Participant 11; F, 63 years)

‘People are not the same, other health care workers treat us well and others don’t. I never received any advice regarding the control of my diabetes.’ (Participant 02; F, 65 years)

All the participants remembered the type of medication they were using and some of them brought out their clinic cards for the interviewer to check the name of the medication. All of them were either on oral medication (metformin, glibenclamide or gliclazide) alone or a combination of oral medication and injections (insulin).

### Theme 2: Factors influencing the control of diabetes

All the participants considered poverty as an important reason why blood sugar is not controlled. Some of the participants explained how lack of money was contributing to poor control of their blood sugar:

‘No money for taxi, hence, cannot keep clinic appointments and cannot go to clinic to fetch my pills.’ (Participants 06; F, 54 years and 08; F, 45 years)

‘I don’t eat breakfast and I cannot drink pills on an empty stomach, sometimes, no money to buy food at home.’ (Participant 02; F, 65 years)

Lack of money is linked to the dietary adjustment required for the control of their blood sugar, which seemed impossible because the majority of the diabetic patients were very poor. They had no money to buy a glucometer to monitor blood sugar at home.

Adherence to medication was explored: thirteen participants (*n* = 13/17) acknowledged that they miss some doses of their medication when they travel away from their home. Six participants reported poor adherence to treatment. Some of the reasons for non-adherence included: forgetfulness in taking medication, not collecting medication from the clinics, fear of taking medication on an empty stomach, being tired of using drugs every day, too many pills to take every day, side-effects of the medication and lack of information:

‘I do not take my medications when going away from home.’ (Participant 16; F, 72 years)

‘I do not think I should used the medication every single day though I sometimes feel weak when not taking them.’ (Participant 07; M, 66 years)

The relationship between diabetes and obesity was explored to gain an understanding of the control of blood sugar. Fifteen participants acknowledged that there is a definite relationship between diabetes and obesity. Some of the participants made reference to themselves as being obese and suggested that that could be the reason for their sugar not being controlled. Some of the participants however, disagreed with the idea of obesity as a probable cause for uncontrolled diabetes.

‘Diabetes does not want fatty food and now when one is obese, it's a sign that you eat more than normal and you also take fatty and unhealthy food so that can make it difficult for your diabetes to be controlled.’ (Participant 10; F, 68 years)

‘There is no relationship between obesity and control of diabetes at all, look at me how tiny and poor I am and it is difficult to control my diabetes.’ (Participant 02; F, 65 years)

Assessment of the quality of diabetes education at the clinics was explored; this generated mixed results. Eight participants were appreciative of the diabetic education provided by the doctors and nurses whilst some were disappointed that doctors were always in a hurry to prescribe medication for the patients. Some of the participants, who reported that health care workers do counsel them about control of their diabetes, felt that the advice was not practical. They were often told to eat healthy, avoid fatty meals, and eat fruit and vegetables, all of which require money to buy, and they are poor. But all of the participants were in agreement that alcohol and cigarette smoking were not good for diabetic patients:

‘No advice from the health care workers, sometimes, they are too much hurrying.’ (Participant 06; F, 54 years)

‘No advice from the health care workers regarding the control of my sugar, I only hear from other people outside who have experience in living with diabetes.’ (Participant 11; F, 63 years)

‘I once visited a private doctor who told me that the combination of the pills is not correct for me but the nurses at the clinic kept on giving the wrong pills.’ (participant 16; F, 72 years)

Fifteen of the participants (*n* = 15/17) reported that their nearest clinics usually do not have doctors; they are compelled to travel to Mthatha to see doctors, which requires money for transport fares. They usually get to the hospital by 06:00 to join the queue for doctors, who would arrive at the consulting rooms at 09:00. Four participants reported that they sometimes see doctors who do outreach programmes at their local clinics:

‘I am dependent on the doctor who visits from the hospital. Absence of doctors at the local clinics creates complications for me, I feel weak when I am exposed to the sun.’ (Participant 07; M, 66 years)

### Theme 3: Perspective of participants on how to improve glycaemic control

All the participants were certain about healthy eating: avoidance of fatty meals, eating small amounts of food at a time and eating fruit and vegetables. Ten participants understood that exercise is beneficial. Six of them would recommend exercise to other diabetic patients. Fourteen participants suggested that diabetic patients must keep their clinic appointments for check-ups. Thirteen participants emphasised that taking medication as directed by the health care workers is crucial for control of blood sugar.

Twelve participants were certain that if people could take their treatment and avoid starvation, their blood sugar would be controlled. Ten of the respondents recognised that diabetic patients need to heed the advice of the health care workers and other people who are living with diabetes. Nearly all the participants (*n* = 16/17) recommended that health centres need to be upgraded to provide quality diabetes care services.

Doctors were needed at each of the local clinics close to their communities; this would relieve the burden of traveling to town to attend hospitals. Dedicated nurses at the clinics should provide diabetes education:

‘No seminars or health educational classes are taking place at the clinics. Government only focuses on HIV/AIDS and other conditions like diabetes are ignored.’ (Participant 01, F, 56 years)

Eleven respondents were convinced that if more attention was diverted towards diabetes care at the various health facilities, patients would achieve better control. The participants reiterated that increased awareness and communication are crucial for improvement in the control of diabetes:

‘Government should try means exactly the way they do with HIV/AIDS. People with HIV/AIDS used to take many pills and now they are only taking one pill.’ (Participant 09; M, 51 years)

Ten participants were of the opinion that government needs to provide money and food supplements to diabetic patients:

‘The government can also try and have a way of identifying those people who are struggling financially and support them with food parcels because sometimes they default as a result of not having food in time so that they can take their medications.’ (Participant 04; M, 53 years)

Fifteen participants thought that by monitoring the blood sugar at home, they would achieve better control, hence suggesting that government should provide glucometers. Some participants also suggested that health facilities must keep a stock of medication for diabetes to prevent running out.

## Discussion

There have been mixed reports^8,14,15,16,17,18,20^ from previous research studies: whilst some reports showed that improvement of knowledge of diabetes care might predict good glycaemic control,^8,14,15^ others did not link with improvements.^11,16,20^ Our study reported that the majority of the respondents (*n* = 15/17) had some knowledge of diabetes and its complications despite their critically poor glycaemic control. Most of the participants (*n* = 14/17) understood what is necessary for good glycaemic control but had little idea about the treatment target for such control. This is an aspect of the diabetes care that clinicians could improve during consultations.

Diabetic patients should have basic knowledge of the treatment goal and what is necessary to achieve this. Basic knowledge of diabetes is considered a prerequisite for self-care management.^11^ Every diabetic patient should, at a minimum, know about the disease condition, complications, treatment options and dietary adjustment. This concept is supported by earlier studies, which suggested that patients with chronic diseases who are engaged and are active participants in their health care have better health outcomes.^14,15^

The finding of poor glycaemic control amongst the participants, despite their good awareness of diabetes and its complications, suggests gaps in translating knowledge to actions. Santos et al.^11^ reported that glycaemic control does not correlate with knowledge of diabetes. They suggested that theoretical understanding of diabetes is not by itself significantly associated with appropriate glycaemic control. Also, Heisler et al.^16^ concluded that the knowledge of HBA1c alone was not sufficient to translate increased understanding of diabetes care into improvement in self-management of diabetes.

Thirteen participants in the study demonstrated poor knowledge of the treatment target for their diabetes. The study by Iqbal et al.^19^ found that the majority (59.5%) were unfamiliar with HBA1c. Knowledge of glycaemic control was generally poor amongst the participants, especially T2DM patients. Intervention with diabetic education, however, yielded improvement in glycaemic control amongst the poorly controlled T2DM, who were in the unfamiliar group. Whether diabetes education would lead to improvement in glycaemic control in this study population requires another research study.

The glycosylated haemoglobin of 9.4% – 13.6% in this study may suggest a poor relationship between diabetes knowledge and glycaemic control. A number of studies have found no significant association between the level of knowledge of diabetes and glycaemic control.^11,20^ However, the evidence for the benefits of patient education on diabetes is overwhelming.^8,10,12,14,15,21^ Hence, national and international bodies continue to emphasise in their recommendations that diabetic education should be provided at all levels of care.^10,21^

Diabetes education of patients should address adherence issues and the other factors of glycaemic control in each patient. Successes from adherence counselling provided to HIV positive individuals^25,26^ might lead to improvement in the key performance indicators of diabetes care. As reported by some of the participants, reasons for poor adherence are many and varied; therefore, adherence counselling of diabetic patients may produce similar results as seen in HIV care. The participants highlighted a number of barriers to achieving good glycaemic control: poverty and its impact on the dietary requirements of diabetes, poor treatment adherence, lack of knowledge of treatment targets and lack of doctors at the primary health care centres in the rural areas. These are in keeping with the determinants of glycaemic control documented in the literature.^27,28,29.30,31,32^

The challenges faced by the study population reflect the level of unemployment, rural dwelling and lack of knowledge of glycaemic control in South Africa. The demand for food parcels and financial support by participants in the study reflects the current economic situation in most rural communities. Patients need money to take taxis, buy food and provide the basic needs of life.

## Limitations

This study is limited by the fact that qualitative study findings cannot be generalised. The selection of more than one health facility for the study might shed more light on the issues of poor glycaemic control in different areas in the country. Future studies should explore the perspectives of health care workers and health managers on glycaemic control.

## Conclusion

The understanding of the patients’ perspectives on the challenges of poor glycaemic control is relevant. Useful data on the overall knowledge of diabetic patients were obtained, as well as the barriers to achieving good glycaemic control. Participants in the study highlighted some of the shortcomings of consultations with clinicians: not spending quality time with patients and not paying proper attention to the particularities of each patient. Availability of doctors in the rural health facilities remains a challenge to equitable health service delivery in South Africa. The re-engineering of primary health care in the country should prioritise health service delivery to the rural communities. The participants in the study provided insight into the probability of an association between poverty and poor glycaemic control. However, the qualitative nature of the study does not allow for such a conclusion to be drawn, hence a prospective study is proposed to test this hypothesis.
